# Fabrication and In Vivo Evaluation of In Situ pH-Sensitive Hydrogel of Sonidegib–Invasomes via Intratumoral Delivery for Basal Cell Skin Cancer Management

**DOI:** 10.3390/ph18010031

**Published:** 2024-12-30

**Authors:** Maha M. Ghalwash, Amr Gamal Fouad, Nada H. Mohammed, Marwa M. Nagib, Sherif Faysal Abdelfattah Khalil, Amany Belal, Samar F. Miski, Nisreen Khalid Aref Albezrah, Amani Elsayed, Ahmed H. E. Hassan, Eun Joo Roh, Shaimaa El-Housiny

**Affiliations:** 1 Department of Pharmaceutics and Drug Manufacturing, Faculty of Pharmacy, Modern University for Technology and Information, Cairo 11435, Egypt; 2 Department of Pharmaceutics and Industrial Pharmacy, Faculty of Pharmacy, Beni-Suef University, Beni-Suef 62511, Egypt; 3 Department of Pharmaceutics and Pharmaceutical Technology, Faculty of Pharmacy, Deraya University, Minya 61768, Egypt; 4 Department of Pharmacology and Toxicology, Faculty of Pharmacy, Misr International University, Cairo 11435, Egypt; 5 Pharmacology Department, Faculty of Medicine, Beni-Suef University, Beni-Suef 62511, Egypt; drsherifpharma@yahoo.com; 6 Department of Pharmaceutical Chemistry, College of Pharmacy, Taif University, Taif 21944, Saudi Arabia; 7 Pharmacology and Toxicology Department, College of Pharmacy, Taibah University, Madina 42278, Saudi Arabia; 8 Department of Obstetric & Gynecology, College of Medicine, Taif University, Taif 21944, Saudi Arabia; 9 Department of Pharmaceutics and Industrial Pharmacy, Faculty of Pharmacy, Taif University, Taif 21944, Saudi Arabia; 10 Department of Medicinal Chemistry, Faculty of Pharmacy, Mansoura University, Mansoura 35516, Egypt; 11 Chemical and Biological Integrative Research Center, Korea Institute of Science and Technology (KIST), Seoul 02792, Republic of Korea; 12 Division of Bio-Medical Science & Technology, University of Science and Technology, Daejeon 34113, Republic of Korea; 13 Department of Pharmaceutics and Drug Manufacturing, Faculty of Pharmacy, Modern University for Technology and Information (MTI), Cairo 11435, Egypt

**Keywords:** basal cell skin cancer, sonidegib, invasomes, chitosan, bioavailability, targeting

## Abstract

Background/Objectives: Basal cell skin cancer (BCSC) develops when skin cells proliferate uncontrollably. Sonidegib (SDB) is a therapeutic option for the treatment of BCSC by inhibiting hedgehog signaling. The problems with SDB’s low solubility, poor bioavailability, resistance, poor targeting, and first-pass action make it less effective when taken orally. This investigation set out to design an intratumoral in situ pH-sensitive hydrogel of SDB-invasomes (IPHS-INV) that can effectively treat BCSC by improving SDB’s bioavailability, sustainability, targeting, and efficacy while also reducing its resistance and undesirable side effects. Methods: Numerous S-INV formulations were developed using Box–Behnken Design Expert and tested before settling on the optimum S-INV formulation. An experimental 7, 12-dimethylbenzanthracene (DMBA) carcinoma rat model was used for in vivo studies of the IPHS-INV formulation after it was combined with chitosan. Results: Phospholipids (1.72% *w*/*w*), cholesterol (0.15% *w*/*w*), ethanol (1% *v*/*v*), and cineole (1.5% *v*/*v*) were shown to be the optimal components in the SDB-invasome formulation. The IPHS-INV formulation outperformed the permeation and bioavailability of free SDB by 7.14 and 6 times, respectively, and sustained its release by 57.41%. The IPHS-INV formulation showed a decrease in tumor volume of 99.05% and a reduction of hypercellular tumors, indicating its anti-cancer activity. The intratumoral IPHS-INV formulation maintained a higher concentration of SDB in tumors, indicating its targeting activity. Conclusions: These findings support the use of the intratumoral IPHS-INV formulation as an effective strategy for the treatment of BCSC.

## 1. Introduction

A neoplastic disease, skin cancer develops when skin cells proliferate uncontrollably [1,2]. The majority of skin cancer diagnoses in the USA are classified as basal cell skin cancer (BCSC) [3,4]. Its incidence is rapidly increasing to over 100,000 per year in the USA, making it one of the main causes of cancer-related mortality [5,6]. White people are facing a major threat from the rising incidence of BCSC, which has been increasing at a rate of 3–8% each year on average [5,6]. The rising incidence of BCSC highlights the need for more effective, secure, and cost-effective cancer treatment. A wide variety of methods have been developed for the treatment of BCSC, including radiation therapy, cryosurgery, photodynamic therapy, and surgical excision [7,8]. Nevertheless, there are significant drawbacks to these procedures that should be considered, such as scarring, the chance of recurrence, infection, a lengthy recovery time, and an effect on healthy tissue [8]. New insights into the function of hedgehog signaling in BCSC development, along with the discovery of hedgehog inhibitors, have created opportunities for therapeutic alternatives that have better clinical results [9,10]. The hedgehog signaling system controls the processes of cell division, tissue regeneration, and maturation [9,10]. As a result, the primary mechanism of action for several anti-cancer drugs is the inhibition of hedgehog functionality [11]. Sonidegib (SDB) is a prominent anti-cancer medication utilized in chemotherapy regimens for the treatment of BCSC [9,10,11]. By selectively inhibiting the smoothening of the positive regulator, SDB disrupts the hedgehog pathway and inhibits tumor growth [9,10,11].

Oral administration of SDB is rendered ineffective due to its limited solubility, resistance, significant side effects, and poor absorption and bioavailability [12,13]. Biological obstacles, high recurrence rates, patient noncompliance, and other issues can also impact other traditional drug delivery techniques, such as transdermal delivery systems or local targeting formulations [8]. To tackle these concerns, intratumoral delivery of nanocarrier-encapsulated medicines (IDNEM) was suggested as an alternative strategy. IDNEM has undergone a significant increase in research over the past decade, with an approximate 1.5-fold rise [14,15]. As IDNEM is directly administered into tumor tissues, it can circumvent trapping by reticuloendothelial systems and enhance their accumulation within tumors [14,15]. Furthermore, unlike free drug injection, the application of IDNEM can inhibit the rapid dissipation and washout of medications from tumor tissues into the bloodstream and adjacent normal tissues by regulating their diffusivity [16]. Accordingly, an in situ pH-sensitive hydrogel of an SDB-invasome (IPHS-INV) formulation has been developed for intratumoral delivery with the aim of improving SDB’s solubility, bioavailability, targeting, and efficacy while simultaneously decreasing its toxicity and release as a possible treatment for BCSC.

The intratumoral system is an intriguing potential alternative to systemic toxicity-reducing chemotherapy for BCSC [17,18]. It has the capacity to enhance the targeting and effectiveness of therapeutics by controlling drug delivery to cancer cells and attaining the required therapeutic concentration of medications at the tumor site [19,20,21]. Increased drug residence time and sustained release of SDB over a longer period of tumor exposure are two possible benefits of the in situ pH-responsive hydrogel for improved drug absorption [22,23]. Chitosan has been used in many investigations due to its pH-sensitive properties because it maintains its liquid form at low pH and its gel at high pH [22,23,24,25]. The mucoadhesive properties, lack of toxicity, and biodegradability of chitosan make it an ideal drug delivery vehicle for both hydrophilic and hydrophobic compounds [22,23,24,25].

One type of nanoparticle is the invasome, which consists of liposomal vesicles that include ethanol and terpenes to increase their penetration [26,27]. They can boost the drug’s bioavailability, permeation, efficacy, and targeting [26,27]. They serve as a depot for sustained delivery of drugs [26,27,28,29]. Ethanol and terpenes are potent penetration enhancers that increase medication absorption by lipid packing disruption and/or bilayer stacking disturbance [26,27,28,29]. Terpenes and ethanol collaborate to increase the medicine’s permeability [26,27,28,29]. This investigation set out to design a formulation of IPHS-INV for intratumoral delivery with the aim of improving SDB’s solubility, bioavailability, permeation, targeting, and efficacy of SDB while also reducing its adverse effects and sustaining its release as a possible treatment for BCSC. A number of different SDB-invasome (S-INV) formulations were developed using the Design Expert software and tested before settling on the optimum S-INV formulation. A 7, 12-dimethylbenzanthracene (DMBA)-induced skin cancer model was used to assess the efficacy, bioavailability, and toxicity of the IPHS-INV formulation after it was combined with chitosan.

## 2. Results and Discussion

### 2.1. Experimental Design

For the purpose of preparing S-INV formulations, the independent variables were selected based on literature surveys and preliminary experiments [26,27,30]. Table 1 shows that different invasomal formulations were effectively created. Invasome consists of liposomal vesicles that include ethanol and terpenes to increase their penetration [26,27]. Prior research assessed the potential risks of administering invasome-forming components to certain organs [31,32,33]. It has been proven that ethanol is a safe and non-irritating penetration booster [33]. Terpenes are typically thought of as harmless and non-irritating due to their nature as naturally occurring substances [31,32]. Also, in vivo tests were conducted on animals to determine the safety of the S-INV formulation. The outcomes that were monitored included mortality, changes in hematology, fluctuations in body weight, and renal and liver dysfunction. Phospholipids are lipid particles that act as transporters for drugs [34]. Cholesterol strengthens vesicular membranes and increases medication retention by bridging gaps created by insufficient packing of other lipid species [34]. Preliminary research indicated that the cholesterol concentration positively affected the entrapment efficiency (EE) and vesicle size (VS). An 11.57% rise in EE and a 29.23% increase in VS were seen upon increasing the cholesterol concentration from 0.05% to 0.3% because it increased the lipophilicity of the lipid bilayer membrane. Nevertheless, the findings indicated that the nanovesicle formulation with cholesterol (0.15% *w*/*v*) was the most efficient. A 6.98% increase in EE and a 12.85% increase in vs. were seen upon increasing the cholesterol concentration from 0.05% to 0.15%. However, a 4.28% increase in EE and a 14.51% increase in vs. were seen upon increasing the cholesterol concentration from 0.15% to 0.3%. These findings were validated by the research of Salem et al. [35]. Following these findings, various S-INV formulations, including phospholipid (1–5%), ethanol (1–5%), cineole (0.5–1.5%), and cholesterol (0.15%), have been developed.

### 2.2. Evaluation and Optimization of S-INV Formulation

#### 2.2.1. Design Expert Software

**Figure 1:**
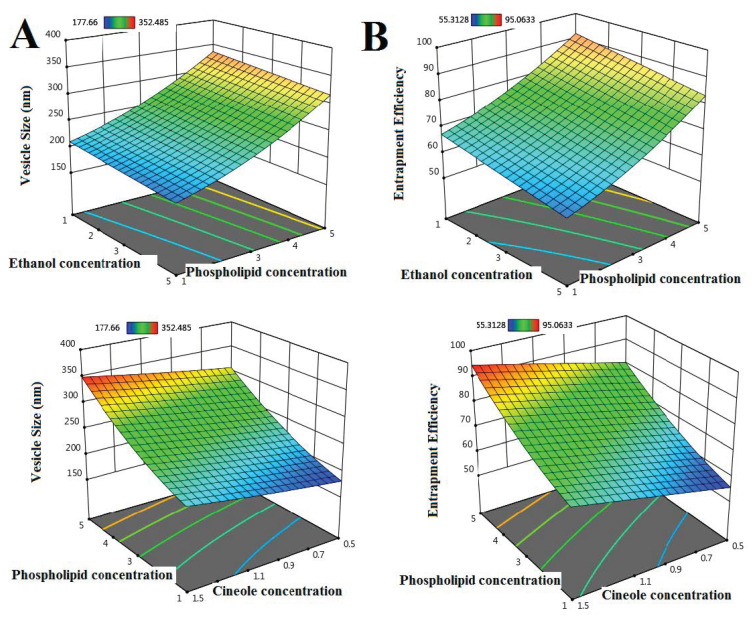
Three-dimensional response surface plot for the effect of concentration of ethanol, phospholipid, and cineole on vs. (**A**) and EE (**B**).

Once the data were collected, the Design Expert software used an analysis of variance (ANOVA) test to find the best-fitting model [36]. To assess if a model provides sufficient explanations for the data, the program calculates many metrics, including probability value (*p*-value), lack of fit, Fisher’s ratio (F-value), predicted determination coefficient (R^2^), adjusted R^2^, and adequate precision. The model is deemed to have enough accuracy for optimization purposes if the p-value is lower than 0.05, the lack of fit is negligible, the F-value is maximum, the predicted and adjusted R^2^ values are the maximum, the difference is less than 0.2, and the adequate precision is more than 4 [36,37]. The data analysis in Table 2 led to the selection of the quadratic model for EE and VS. Coded polynomial Equations (3) and (4) and 3D surface graphs (Figure 1) are also provided by the program to help in interpreting the model and determining the effect of each variable [25,37].

#### 2.2.2. Vesicle Size (VS) Assessment

Minimizing vs. appears to improve drug absorption and bioavailability [38,39]. Investigations were carried out to determine the optimum ethanol (A), phospholipid (B), and cineole (C) concentrations. Figure 1A demonstrates that augmenting the phospholipid content (B) had a positive effect on the VS. After going from a 1% (F4) concentration of phospholipids to a 5% (F1) concentration, the rise in vs. was 65.65% because it produces thicker phases and higher mass transfer resistance. Fouad et al.’s research confirmed these findings [34].

The vs. of the S-INV formulations was negatively (*p*-value < 0.05) correlated with the ethanol concentration (A); for example, F3 and F1 (5% ethanol) had a vs. that was 7.04% and 6.70% lower than F14 and F12 (1% ethanol), respectively. An increase in ethanol concentration causes a decrease in vesicle volume and membrane thickness. When ethanol goes through hydrocarbon chains, it changes the vesicles’ net charge, which makes them smaller. These findings corroborate Abou-Taleb et al. [40].

The vs. of S-INV formulations was shown to be directly (*p*-value < 0.05) proportional to the cineole concentration (C) because of the increased demand for packing on the surface area of the membrane and the disruption of the packing of the lipid bilayer. For example, F2 and F3 (0.5% cineole) had vs. that was 22.57% and 18.92% greater than that of F8 and F5 (1.5% cineole), respectively. El-Tokhy et al. also found results that were similar to these [39].

VS (nm) = +249.49 − 9.62A + 63.06B + 20.78C − 0.3588AB + 0.1137AC + 0.2363BC + 0.0685 A^2^ + 13.86B^2^ + 1.63C^2^ (1)

#### 2.2.3. Entrapment Efficiency (EE) Analysis

Figure 1B demonstrates that augmenting the phospholipid content had a positive effect on the EE. After going from a 1% (F4) concentration of phospholipids to a 5% (F1) concentration, the rise in EE was 43.53% because it encourages the formation of strong and cohesive layers that surround the drug in vesicles. Fouad et al.’s research confirmed these findings [34].

The EE of the S-INV formulations was shown to be inversely (*p*-value < 0.05) proportional with the ethanol concentration; F14 and F10 (containing 1% ethanol) outperformed F3 and F4 (containing 5% ethanol) by 14.85% and 13.37%, respectively, in terms of EE. This occurs because higher amounts of ethanol have the ability to break down phospholipids, leading to the bilayer becoming porous and leaking. Kamran et al.’s research confirmed these findings [41].

The EE of the S-INV formulations decreased as the cineole level increased; for example, F8 and F9, which included 1.5% cineole, have an EE that was 24.58% and 14.92%, respectively, higher than F2 and F13, which contained 1% cineole. This is due to the fact that larger concentrations of cineole have the ability to dissolve lipophilic medicines like SDB, allowing them to penetrate the lipid bilayer and enhance its EE. These results align with those of Ahmed et al. [38].

EE (%) = +72.64 − 4.44A + 12.50B + 6.87C + 0.1128AB + 0.0389AC + 0.1018BC + 0.0328 A^2^ + 2.45B^2^ − 0.0570C^2^ (2)

#### 2.2.4. Optimization of S-INV Formulation

Following the application of EE and vs. constraints, the Design Expert program suggested an optimum S-INV formulation. Phospholipids (1.72% *w*/*w*), cholesterol (0.15% *w*/*w*), ethanol (1% *v*/*v*), and cineole (1.5% *v*/*v*) were shown to be the optimal components in the S-INV formulation. In terms of mean VS, the optimum S-INV formulation measured 246.49 ± 2.41 nm and an average EE of 76.92 ± 0.40%. The observed EE and vs. of the optimum S-INV formulation were highly concordant with the predicted values, indicating that the optimization approach was appropriate and valid.

### 2.3. Assessment of Optimum S-INV Formulation

#### 2.3.1. Homogeneity and Physical Stability Evaluation

The polydispersity index (PDI) allows one to learn about the dispersion and consistency of vesicle sizes [34]. A homogenous vesicle population and a formulation with a significantly monodispersed structure are suggested by an optimum PDI of 0.30 ± 0.05 for the optimum S-INV formulation.

The physical stability and absence of aggregation tendency of the optimum S-INV were demonstrated by its zeta potential, which was (−) 26.0 ± 2.1, according to the results. Phospholipid and ethanol content may account for these results; these compounds reduce the possibility of vesicular charge de-aggregation and stabilize it by electrostatic attraction.

#### 2.3.2. Differential Scanning Calorimetry (DSC)

**Figure 2:**
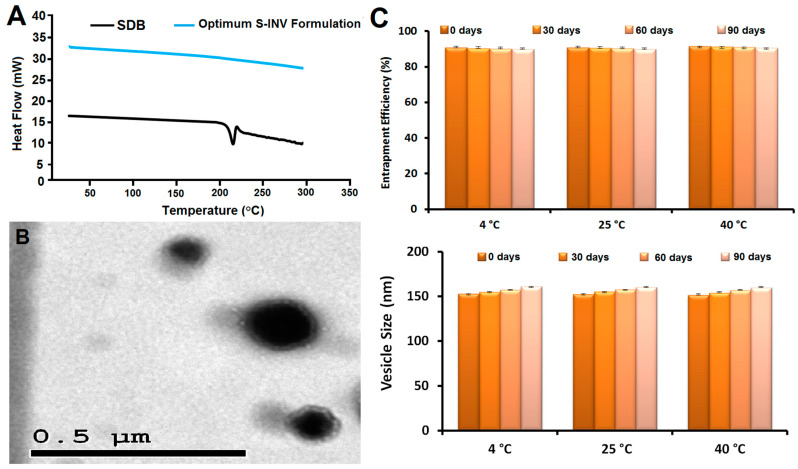
(**A**) DSC thermograms of optimum sonidegib–invasome (S-INV) formulation; (**B**) TEM image of optimum S-INV formulation; (**C**) stability study of the S-INV formulation at 4 °C, 25 °C, and 40 °C.

The optimum S-INV formulation and the DSC thermograms of the SDB are displayed in Figure 2A. At its melting point of 214.5 °C, the SDB’s thermogram of SDB revealed a distinct endothermic peak. The SDB’s peak in the thermogram of the optimum S-INV formulation disappeared, indicating that SDB was entirely integrated after existing in an amorphous state, and lipid bilayers entrapped SDB more efficiently.

#### 2.3.3. Transmission Electron Microscopy (TEM)

See Figure 2B for the results of the TEM investigation into the morphology of the optimal S-INV formulation. The spherical vesicles, which did not agglomerate, were easily discernible as tiny black dots distributed uniformly across the nanoscale.

#### 2.3.4. Stability Evaluation

The S-INV formulation’s stability was undertaken in order to ascertain the best way to keep it for maximum effectiveness [35]. Figure 2C shows that the optimum S-INV formulation did not change substantially in EE or vs. after three months of storage at 4 °C, 25 °C, and 40 °C (*p*-value > 0.05). Consequently, the optimal S-INV formulation exhibited stability at 4, 25, and 40 °C.

### 2.4. Fabrication and Evaluation of IPHS-INV Formulation

To achieve desirable rheological and mucoadhesive properties, chitosan is utilized to enable the sustainable delivery of medications [42,43]. To establish the chitosan concentration, we carried out a literature review and preliminary studies [24,25]. Due to its high viscosity, lack of irritation, and appropriate rheological properties, chitosan finds widespread application [24,25]. The fact that a chitosan solution maintains its liquid state at low pH and its gel state at high pH suggests it could be an excellent carrier for creating a pH-responsive hydrogel [23,44]. Chitosan at a concentration of 0.67% was chosen for its ability to make an in situ pH-sensitive hydrogel that is both more stable and elastic. Our research aligns with Salem et al. [25]. Consequently, an in situ pH-sensitive hydrogel was produced by converting free SDB and the optimum S-INV formulation with 0.67% chitosan.

#### 2.4.1. pH Analysis

When planning the application of the in situ pH-sensitive hydrogel formulation, it is crucial to consider its pH [43]. The absence of irritation was confirmed by the pH values of 5.4 ± 0.31 for free SDB in situ pH-sensitive hydrogel and 5.3 ± 0.27 for the IPHS-INV formulation.

#### 2.4.2. Viscosity Study

In comparison to free SDB in situ pH-sensitive hydrogel and optimum S-INV, the IPHS-INV formulation exhibited a 2.74-fold and 5.23-fold increase in viscosity, respectively (Table 3), with a *p*-value < 0.05. The optimum S-INV formulation had a much lower viscosity than the IPHS-INV formulation, which was caused by the presence of the chitosan polymer. Invasomes’ phospholipid content causes the creation of thick bilayers, which in turn causes the IPHS-INV formulation to have a much greater viscosity compared to free SDB in situ pH-sensitive hydrogel. When designing in situ gels for injection, syringe-ability is a key consideration since it determines how easy the gels will be to inject into the body [45,46]. Syringe-ability is directly related to the viscosity coefficient [47]. To be considered syringe-able, a viscosity coefficient of approximately 300 cP is required [45,46,47]. The viscosity coefficient of the IPHS-INV formulation was found to equal 251.25 ± 7.16 cP, indicating its suitability for syringe-ability and injectability.

#### 2.4.3. Ex Vivo Permeation Study

**Figure 3:**
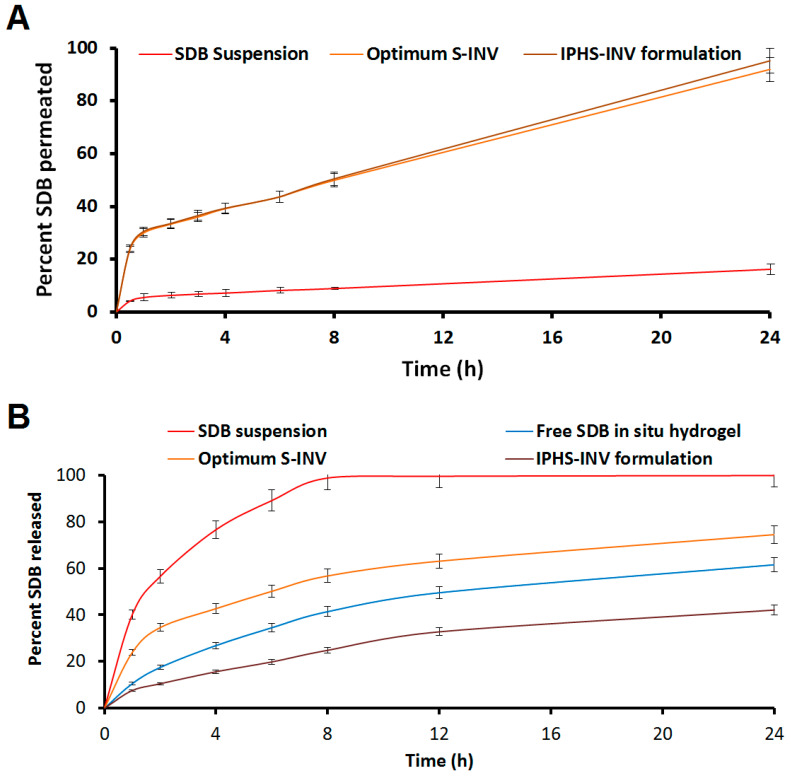
(**A**) Permeation profile of IPHS-INV formulation; (**B**) Release profile of IPHS-INV formulation. Data denote mean ± SD (n = 3).

The IPHS-INV formulation’s ex vivo permeability characteristics are compared to those of free SDB in situ pH-sensitive hydrogel, free SDB suspension, and optimum S-INV in Figure 3A. When compared to free SDB suspension and free SDB in situ pH-sensitive hydrogel, the percentage of SDB permeation from optimum S-INV and IPHS-INV formulation was substantially greater (*p*-value < 0.05). Table 3 shows that the optimum S-INV formulation outperformed the free SDB suspension by a factor of 6.42 and 6.82, respectively, for permeation and steady-state flux (Jss). An analysis with a *p*-value < 0.05 revealed that the IPHS-INV formulation outperformed the free SDB suspension in terms of both permeation at 6.65 times and Jss at 7.14 times. With a *p*-value < 0.05, the IPHS-INV formulation outperformed the free SDB in situ pH-sensitive hydrogel in terms of both permeation with 6.41 times and Jss with 7.01 times. It is believed that invasomes reach the lipid layers and release terpenes, ethanol, and phospholipids, which enhance penetration. Dragicevic-Curic et al. found the same outcomes [48].

#### 2.4.4. SDB’s Release Kinetics

As shown in Figure 3B, the IPHS-INV formulation exhibited a different in vitro SDB release profile compared to free SDB suspension, optimum S-INV, and free SDB in situ pH-sensitive hydrogel. The prolonged-release pattern of SDB was shown by the significantly reduced percentage release of SDB from optimum S-INV and IPHS-INV formulations compared to free SDB suspension (*p*-value < 0.05). Within eight hours, nearly all of the SDB had been released. Table 3 demonstrates that compared to the free SDB suspension, a 24.62% decrease (*p*-value < 0.05) in SDB’s release was seen in the optimum S-INV formulation, and a 57.41% decrease (*p*-value < 0.05) in SDB’s release was seen in the IPHS-INV formulation. These results might be due to the presence of cholesterol and phospholipids. The slow release of the IPHS-INV formulation is explained by the presence of chitosan polymer.

Based on a criterion that takes into account the highest R^2^ and model selection criterion (MSC) values as well as the lowest Akaike information criterion (AIC), the DDsolver program chooses the Korsmeyer–Peppas model to best fit the IPHS-INV formulation. The release of SDB from the IPHS-INV formulation was facilitated by non-Fickian diffusion, as indicated by the calculated Korsmeyer–Peppas coefficient “n” value of 0.691. The sustained release pattern of the IPHS-INV formulation was verified through the use of mean dissolution time (MDT). The IPHS-INV formulation’s capacity to retain drugs becomes increasingly noticeable as the MDT value increases [49]. The IPHS-INV formulation’s 3.44 MDT confirmed its prolonged release pattern, in contrast to the free SDB suspension’s 2.32 MDT. A significant variation in the dissolving pattern was seen in the IPHS-INV formulation, with a high difference factor (*f*1) value of 66.60 and a low similarity factor (*f*2) value of 18.54.

### 2.5. In Vivo Assessment of IPHS-INV Formulation

#### 2.5.1. Bioavailability Studies

**Figure 4:**
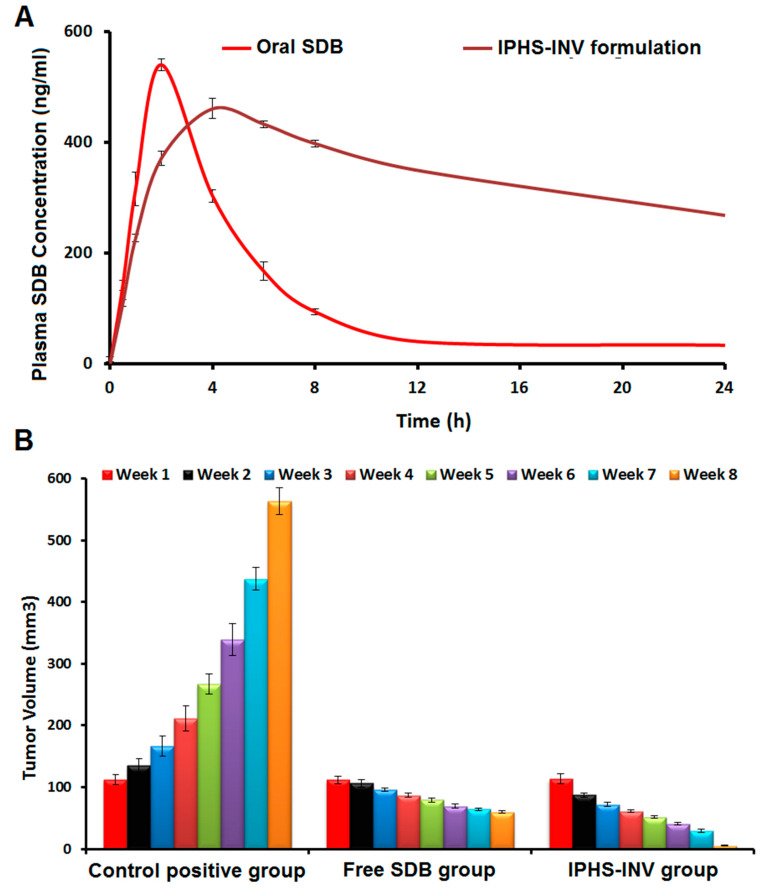
(**A**) Sonidegib (SDB) plasma concentration-time of IPHS-INV formulation. (**B**) Change in tumor volume of IPHS-INV formulation versus the positive control group. Data denote mean ± SD (n = 6).

Our goal was to evaluate SDB’s in vivo pharmacokinetic behavior using HPLC. After giving rats either oral free SDB or intratumoral IPHS-INV formulation, the HPLC method was shown to be both sensitive and specific in detecting the plasma concentration of SDB, as evidenced by the linearity of the SDB regression equation and an R^2^ of 0.998. Using the pKSolver software, we were able to determine the maximum plasma concentration (C_max_), time to reach maximum plasma concentration (T_max_), half-life (t_0.5_), mean residence time (MRT), and area under the curve (AUC) [50], as shown in Table 3. Intratumoral IPHS-INV formulation significantly (*p*-value < 0.05) decreased C_max_ by 12.69% compared to oral free SDB (Figure 4A), suggesting lower unwanted SDB concentration and, hence, less toxicity. T_max_, MRT, and t_0.5_ are sustainability parameters that are utilized to evaluate the formulation [51]. Intratumoral IPHS-INV formulation significantly (*p*-value < 0.05) increased T_max_, MRT, and t_0.5_ by 2-, 5-, and 4.88-fold, respectively, compared to oral free SDB. This suggests that the SDB is sustainable, leading to a slow plasma drug clearance. As a result, there is no need for frequent dosing and fewer adverse effects. At the site of administration, the intratumoral IPHS-INV formulation served as a reservoir, releasing the drug in a controlled manner over time. These findings validated the IPHS-INV formulation’s in vitro release profile.

The relative bioavailability for the intratumoral IPHS-INV formulation was found to be considerably (*p*-value < 0.05) greater than that of the oral free SDB, with a sixfold improvement in AUC0−α as compared to the oral free SDB (Table 4). Cineole and ethanol boosted medication uptake, permeation efficiency, and accessibility to the systemic circulation by increasing drug permeation through the skin, leading to these outcomes. Invasomes have a greater surface area and increased absorption capacity due to their small vesicle size. Results from the bioavailability study were compared with those from similar investigations conducted by Gamal et al. [52]. A transdermal ethosome gel of sonidegib was produced by Gamal et al. to boost its bioavailability. When compared to free sonidegib, the optimal gel formulation had a relative bioavailability that was 3.18 times greater. The synergistic effects of invasomes and chitosan led to more favorable outcomes in our investigation.

#### 2.5.2. Assessment of Effectiveness of Treatment

Research on cancer treatments frequently uses animal models of chemically induced carcinogenesis to evaluate the diagnostic and therapeutic possibilities of novel drugs [53,54,55]. Our goal in this study was to use DMBA to simulate the development of skin cancer [53,54,55]. DMBA is a representative polycyclic aromatic hydrocarbon belonging to a significant category of environmental carcinogens, historically employed for the purpose of tumor promotion in experimental animals [56,57,58]. The application of DMBA to the skin causes free radical generation, which in turn causes the growth of epidermal neoplasia. This process consists of two stages: the irreversible tumor initiation step and the reversible but cumulative tumor-promoting stage [53,54,55]. By increasing the expression of the aryl hydrocarbon receptor in the cell cytosol, DMBA can induce tumors to form in the rat skin [53,54,55]. Many cancer studies utilize DMBA as a chemically induced carcinogenesis in rats as a model because it closely resembles human malignancies in terms of histological features, the sequential growth of tumors, and the activation of ras gene family members [53,54,55,56,57,58,59]. Animals were subjected to DMBA-induced skin cancer experiments. In order to confirm that skin cancer was successfully progressing, we monitored factors like tumor volume, body weight, death rate, and tumor histological features. [57,60].

#### 2.5.3. Tumor Volume

**Figure 5:**
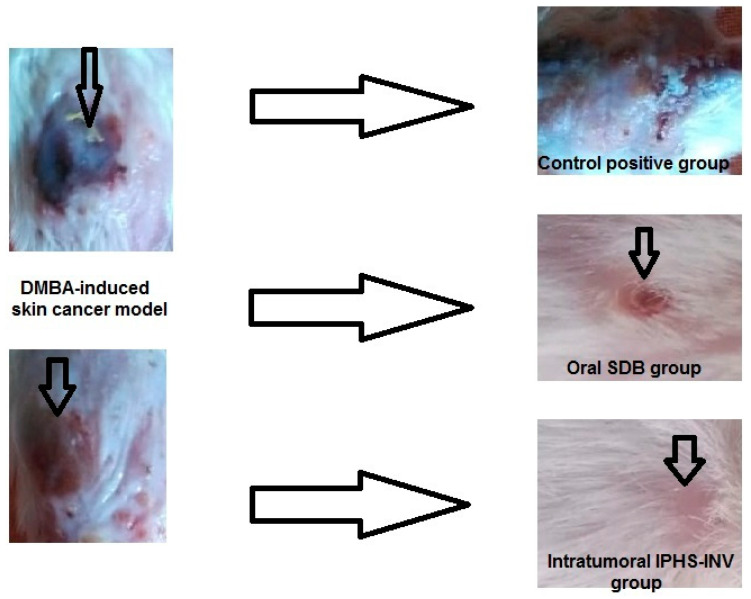
Representative images of DMBA carcinoma in treated rats in the intratumoral IPHS-INV group compared to the DMBA positive control group and oral free sonidegib (SDB) group.

The tumor volume (Figure 4B) in the positive control group rose (*p*-value < 0.05) fivefold at the end of the experiment compared to the beginning, indicating that the rats were successfully inoculated with DMBA-induced skin cancer. Compared to the beginning of the investigation, the tumor volume was significantly (*p*-value < 0.05) reduced by 46.70% in the oral free SDB group and by 95.32% in the intratumoral IPHS-INV group. Figure 4B demonstrates that compared to the positive control group, 89.42% and 99.05% decrease (*p*-value < 0.05) in tumor volume were seen in the oral free SDB and the intratumoral IPHS-INV groups, respectively. Both the intratumoral IPHS-INV and the oral free SDB exhibited anti-cancer activity, according to these research findings. Compared to the oral free SDB group, a 91.09% decrease (*p*-value < 0.05) in tumor volume was seen in the intratumoral IPHS-INV group, which is a greater indicator of its anti-cancer effectiveness. Additionally, Figure 5 shows the tumor size of each group, confirming the effectiveness of the intratumoral IPHS-INV formulation. The reason behind this is that phospholipids, ethanol, and cineole were able to infiltrate the lipid more effectively, leading to more fluidity, diffusion, and flexibility. Improved SDB bioavailability was achieved with the creation of the intratumoral IPHS-INV formulation, which allowed for more penetration and a more constant release rate.

#### 2.5.4. Body Weight Measurement

**Figure 6:**
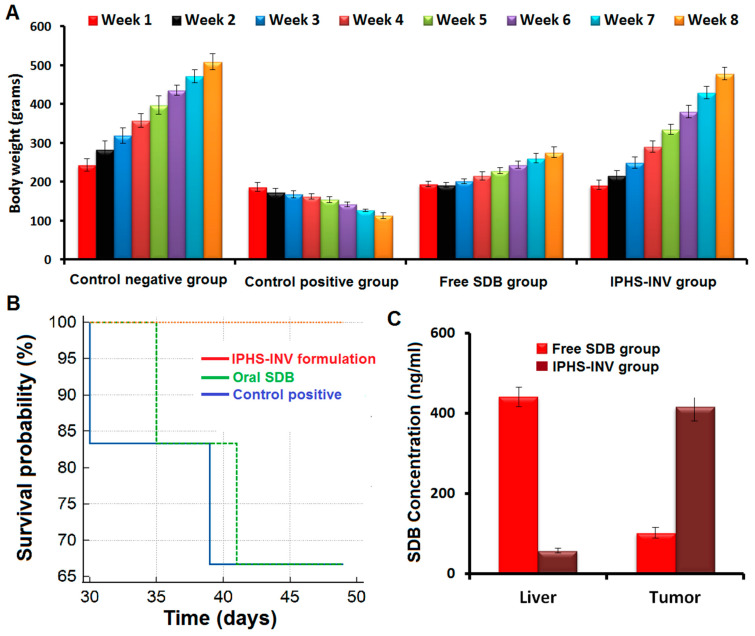
(**A**) Change in body weight of IPHS-INV formulation. Data denote mean ± SD (n = 6). (**B**) Survival probability of IPHS-INV formulation. (**C**) Targeting effect of IPHS-INV formulation.

The body weight (Figure 6A) in the positive control group dropped (*p*-value < 0.05) by 39.29% and 77.73% at the end of the experiment compared to the beginning and the control negative group, respectively, indicating that the rats were successfully inoculated with DMBA-induced skin cancer. Compared to the beginning of the investigation, the body weight was significantly (*p*-value < 0.05) increased by 1.28-fold in the oral free SDB group and by 2.5-fold in the intratumoral IPHS-INV group. Body weight increased (*p*-value < 0.05) by 2.19- and 4.22-fold, respectively, after oral SDB administration and the intratumoral IPHS-INV formulation application, in comparison to the positive control group. Both the intratumoral IPHS-INV formulation and the oral free SDB demonstrated anti-cancer efficacy, as confirmed by these investigations. The intratumoral IPHS-INV demonstrated a 1.93-fold increase (*p*-value < 0.05) in body weight compared to the oral free SDB group, indicating a higher anti-cancer effect.

#### 2.5.5. Mortality and General Behavior

There was a 33.33% increase (*p*-value < 0.05) in mortality (Figure 6B) for the positive control group. Groups that received intratumoral IPHS-INV (49 days) or oral free SDB (44.33 days) had significantly longer mean survival times (MSTs) than the positive control group (42.33 days). Compared to the positive control group, survival time was 4.72 percent longer in the oral free SDB group and 15.7 percent longer in the intratumoral IPHS-INV group, demonstrating the efficacy and safety of the different treatments. Rats in the intratumoral IPHS-INV group showed no signs of behavioral deficits or mortality. Compared to the oral free SDB group, a 10.53% improvement in survival time was seen in the intratumoral IPHS-INV group, indicating that it exhibited greater anti-cancer action.

#### 2.5.6. Histopathology Study

**Figure 7:**
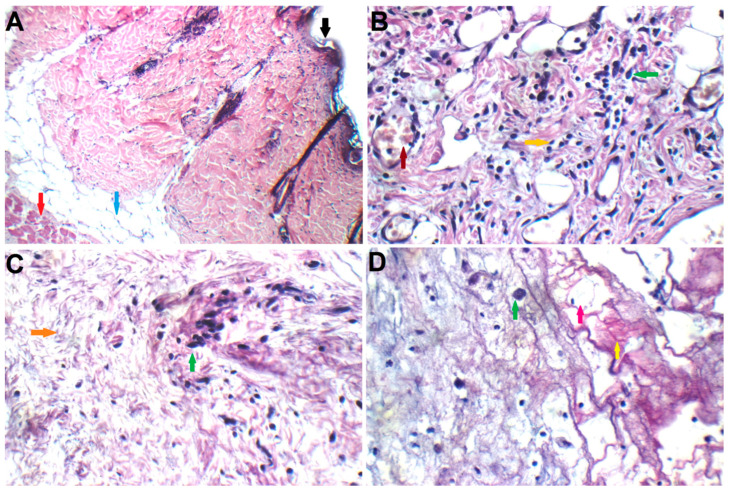
Histopathology images of (**A**) negative control group, (**B**) positive control group, (**C**) oral free sonidegib (SDB) group, and (**D**) IPHS-INV formulation group; black arrow: epidermis; red arrow: muscles; blue arrow: subcutis; green arrow: hypercellular tumor; orange arrow: fibrotic stroma; brown arrow: blood vessels; yellow arrow: necrosis.

Histopathology (Figure 7A) findings from the control negative group showed an intact epidermis with average subcutis and sweat glands. Histopathology (Figure 7B) findings from the positive control group showed a large hypercellular tumor composed of markedly pleomorphic rounded and spindle cells (green arrow) with fibrotic stroma (orange arrow), mildly congested blood vessels (brown arrow), and infiltrating surrounding fat, indicating that the rats were successfully inoculated with DMBA-induced skin cancer.

A small hypocellular tumor made of slightly pleomorphic rounded cells with fibrotic stroma, scattered apoptosis, and non-infiltrating muscles was seen during the histopathological investigation of the oral free SDB group (Figure 7C). A small hypocellular tumor made of slightly pleomorphic rounded cells with an area of necrosis was seen during the histopathological investigation of the intratumoral IPHS-INV (Figure 7D). These results confirmed the efficacy of oral SDB and intratumoral IPHS-INV formulation.

#### 2.5.7. Targeting Study

Based on the results, it was observed that the intratumoral IPHS-INV significantly (*p*-value < 0.05) increased the tumor concentration of SDB (Figure 6C), and the oral SDB significantly increased the liver concentration of SDB. Intratumoral IPHS-INV maintained a higher concentration of SDB in tumors than oral free SDB (*p*-value < 0.05), indicating that intratumoral IPHS-INV could accumulate more SDB in tumor tissues, improving its targeting effect and anti-tumor activity.

#### 2.5.8. Toxicity Studies

Rats of the intratumoral IPHS-INV group showed no observable behavioral deficits or death. It can be concluded that the intratumoral IPHS-INV did not negatively (Figure 6A) impact the animals’ growth and development because, in terms of body weight, the control negative group did not vary significantly (*p*-value > 0.05).

**Figure 8:**
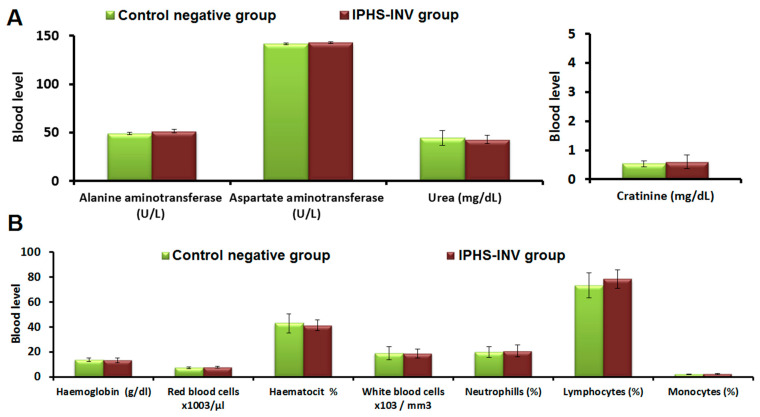
(**A**) Biochemical and (**B**) hematological parameters of the intratumoral IPHS-INV formulation.

The intratumoral IPHS-INV did not negatively (Figure 8A) impact the animals’ renal or hepatic functioning because, in terms of alanine transaminase (SGPT), aspartate transaminase (SGOT), urea, or creatinine, the control negative group did not vary significantly (*p*-value > 0.05).

There were no harmful effects on erythropoiesis and immune systems in animals given intratumoral IPHS-INV because, in terms of hemoglobin, red blood cell count, hematocrit, white blood cells, neutrophils, and monocytes, the control negative group did not vary significantly (*p*-value > 0.05).

As shown in Figure 7D, the intratumoral IPHS-INV group underwent histological examination. There was no inflammatory reaction, reddening, or swelling in the dermal, epidermal, or subcutaneous layers of the skin. These findings support the use of intratumoral IPHS-INV as a viable and effective strategy for the treatment of BCSC.

## 3. Materials and Methods

### 3.1. Materials

Compounds like cholesterol, phospholipid, ethanol, and cineole were acquired from Sigma-Aldrich (St. Louis, MO, USA). Cornell Lab Company (Cairo, Egypt) supplied methanol, chitosan, citric acid, and chloroform.

### 3.2. Preliminary and Optimization Studies

To choose the optimum S-INV formulation, a 3^3^ Box–Behnken design was conducted using Design Expert software^®^ (version 12.0.6.0, StatEase Inc. Minneapolis, MN, USA) to fabricate various SDB-invasome (S-INV) formulations, as shown in Table 1. The independent variables that were defined were the concentrations of ethanol (A), phospholipid (B), and cineole (C). The dependent variables that were defined were the evaluation of vesicle size (VS) and entrapment efficiency (EE).

### 3.3. Preparation of S-INV Formulations

By employing the thin-film hydration process, variations of the S-INV formulations were fabricated [34]. Phospholipid (1–5% *w*/*v*), SDB (10 mg), cineole (0.5–1.5% *v*/*v*), cholesterol (0.15% *w*/*v*), and a methanol–chloroform solution were the components combined. The mixture was mixed in a rotavapor flask set to 100 rpm using a rotary evaporator (Heidolph VV 2000, Burladingen, Germany). It was then allowed to evaporate under reduced pressure in a water bath maintained at 40 °C. The S-INV formulations were then made by spinning 10 mL of a phosphate-buffered solution (PBS, pH 5.5) containing ethanol (1–5% *v*/*v*) at 60 °C for 2 h. The S-INV suspensions were maintained at 4 °C for the purpose of conducting additional analysis.

### 3.4. Characterization of S-INV Formulations

#### 3.4.1. Analyzing EE

Through the use of ultracentrifugation, the entrapment efficiency of several S-INV formulations was assessed [61]. The mixtures were subjected to 2 h of spinning at 15,000 rpm in a centrifuge (Model 8880, W. Sussex, UK) set at 4 °C. Using a UV-vis spectrophotometer (model UV-1601 PC, Tokyo, Japan) at 276 nm and a preset calibration curve, the concentration of SDB was measured in triplicate (n = 3) after collecting the clear supernatant. The formula for calculating the trapped percentage of SDB is as follows:

(3) $$\mathrm{EE}\;\left(\%\right)=\frac{\mathrm{Initial}\;\mathrm{amount}\;\mathrm{of}\;\mathrm{SDB}\;\mathrm{added}\;-\mathrm{amount}\;\mathrm{of}\;\mathrm{free}\;\mathrm{SDB}\;}{\mathrm{Initial}\;\mathrm{amount}\;\mathrm{of}\;\mathrm{SDB}\;\mathrm{added}\;}\times 100$$

#### 3.4.2. Analyzing VS

Each of the developed S-INV formulations had their vs. determined using a Zetasizer instrument (Malvern Instruments, Worcestershire, UK) [62]. Each formulation sample was tested three times (n = 3) at 25 °C after being mixed with an appropriate volume of deionized water (9 mL) for every 1 mL of sample.

#### 3.4.3. Optimization of S-INV Formulations

The findings of each dependent variable were examined using ANOVA in the Design Expert^®^ software. Criteria for determining the best fit for the data included p-value, lack of fit, F-value, adjusted R^2^, predicted R^2^, and adequate precision. In order to find the optimum S-INV formulation, optimization was performed based on the desirability index to determine the minimum vs. while maintaining the maximum EE. Preparing and evaluating the recommended optimum S-INV formulation three times (n = 3) allowed us to confirm the software’s derived factors and predicted responses.

### 3.5. Analyzing the Optimum S-INV Formulation

#### 3.5.1. Homogeneity and Physical Stability Evaluation

The zeta potential is a reliable indicator of vesicle stability, as it quantifies the intensity of the attraction and repulsion involved between them [63]. Vesicle dispersion and homogeneity can be reliably shown by PDI [63]. The zeta potential and PDI of the optimum S-INV formulation were determined using the Zetasizer (Malvern Instruments, Worcestershire, UK) in triplicates (n = 3), in accordance with the previously described method of vs. measurement [62].

#### 3.5.2. DSC

Crystallinity, thermal behavior, and possible interaction of SDB with invasome components were evaluated using the DSC (DSC-50 Shimadzu Corporation, Kyoto, Japan) [35]. A standard aluminum pan was used to compress and heat SDB and optimum S-INV samples at 10 °C/min from 25 to 300 °C, while 100 mL/min of nitrogen was constantly purged.

#### 3.5.3. TEM

Visualization of the optimum S-INV vesicles was accomplished using TEM (H-600, Hitachi, Japan) [34]. The sample was film-applied onto a carbon-coated copper grid, and subsequently, a 1% phosphotungstic acid was used for staining. After allowing the grid to air dry entirely, the samples were analyzed using a TEM.

#### 3.5.4. Stability Studies

Over a period of three months, we monitored changes in vs. and EE at 4 °C, 25 °C, and 40 °C to determine the S-INV’s stability [34]. Monthly, triplicate measurements (n = 3) of vs. and EE were taken.

### 3.6. Fabrication and Evaluation of IPHS-INV Formulation

#### 3.6.1. Fabrication of IPHS-INV Formulation

In citric acid solution, 0.67% *w*/*w* chitosan was dissolved to fabricate the in situ pH-sensitive formulation [25]. To make the IPHS-INV formulation, the optimum S-INV was added to the chitosan solution while stirring gently. To create free SDB in situ pH-sensitive hydrogel, a chitosan solution was gradually mixed with free SDB while swirling gently.

#### 3.6.2. pH Analysis

A pH meter (Griffin, model 80, Britain) was used to measure the pH values (n = 3) of distilled water in which samples of IPHS-INV formulation and free SDB in situ pH-sensitive hydrogel were dissolved [64].

#### 3.6.3. Rheological Study

The viscosity of a hydrogel is a key determinant of its efficiency in circulation [22,23]. The IPHS-INV formulation and free SDB in situ pH-sensitive hydrogel’s viscosity were measured using a Brookfield viscometer (DV-III, AMETEK Brookfield, Middleborough, MA, USA). Switching the shear rate from 0 to 100 s^−1^ and back again allowed us to acquire the necessary measurements. Three independent computations (n = 3) were performed at a temperature of 25 °C to ascertain the viscosity at 10 rpm and the viscosity coefficient of both the IPHS-INV formulation and free SDB in situ pH-sensitive hydrogel [24]. The viscosity coefficient was calculated from the intercept of log *shear rate* against log *shear stress* according to the following equation:

Log shear stress = N log shear rate − log viscosity coefficient(4)

#### 3.6.4. Ex Vivo Skin Permeation Study

The permeation investigations were performed on rat skin using the Hanson dissolving device (Hilab, Düsseldorf, Germany). The equipment exhibited a permeation area of 5 cm^2^ and a receptor cell volume of PBS (50 mL, pH of 5.5) and Tween 80 (0.1% *v*/*v*). The rat underwent surgical removal of subcutaneous tissues and trimming of its fur. To obtain the skin ready to be used as a diffusion membrane, it was washed and submerged in PBS at 4 °C the day before the experiment [34]. Ethics Committee approval (024-013) was obtained from Beni-Suef University. The procedure involved immersing the skin sample in a receptor media and placing it on top of each cell. A known quantity of SDB suspension, optimum S-INV, IPHS-INV formulation, and free SDB in situ pH-sensitive hydrogel equivalent to 2 mg of SDB were included. The Hanson dissolving apparatus was operated with continuous stirring at 100 rpm with a temperature of 32 ± 1 °C. The sink condition was maintained by periodically reintroducing 3 mL of medium into the receptor compartment at different intervals for 24 h. SDB’s concentration was obtained by taking three separate readings (n = 3) at 276 nm with a UV-vis spectrophotometer. The data were presented based on the cumulative amount of SDB that permeated after a 24 h period and the Jss.

(5) $$\mathrm{Ex}\;\mathrm{vivo}\;\mathrm{permeation}\;\left(\%\right)=\frac{\mathrm{The}\;\mathrm{amount}\;\mathrm{of}\;\mathrm{SDB}\;\mathrm{permeated}\;\mathrm{at}\;\mathrm{time}\;t}{\mathrm{Initial}\;\mathrm{amount}\;\mathrm{of}\;\mathrm{entrapped}\;\mathrm{SDB}}\times 100$$

(6) $$\mathrm{Jss}=\frac{\mathrm{The}\;\mathrm{permeation}\;\mathrm{rate}}{\mathrm{The}\;\mathrm{active}\;\mathrm{diffusion}\;\mathrm{area}}\;$$

#### 3.6.5. In Vitro Release Study

An in vitro release study was conducted using the dialysis method [34]. Specified quantities of optimum S-INV, free SDB suspension, IPHS-INV formulation, and free SDB in situ pH-sensitive hydrogel, each containing 2 mg of SDB, were included. The apparatus was comprised of PBS (50 mL, pH of 5.5) and Tween 80 (0.1% *v*/*v*) serving as a releasing medium. Using a temperature of 32 ± 1 °C and continuous stirring at 100 rpm, the Hanson dissolution apparatus was employed. In order to keep the receptor compartment in a sink condition for 24 h, 3 mL of medium was taken out and returned at different times. To find the concentration of SDB, a UV-vis spectrophotometer at 276 nm was used to obtain three readings (n = 3). The data were presented based on the cumulative amount of SDB that was released after a 24 h period.

(7) $$\%\mathrm{Release}=\frac{\mathrm{The}\;\mathrm{amount}\;\mathrm{of}\;\mathrm{SDB}\;\mathrm{released}\;\mathrm{at}\;\mathrm{time}\;t}{\mathrm{Initial}\;\mathrm{amount}\;\mathrm{of}\;\mathrm{entrapped}\;\mathrm{SDB}}\times 100$$

To assess the release kinetics and compare the outcomes to a reference product, DDSolver was employed in Microsoft Excel [65]. To select the best fit model, various statistical metrics were computed. The IPHS-INV formulation’s release mechanism was elucidated by determining the release exponent “n”. The MDT of the IPHS-INV formulation was assessed using DDSolver software to verify its sustainability [49]. The calculation of the *f*2 and *f*1 factors is necessary to find the equivalence of dissolution data. With a *f*2 value of 50 or higher and a *f*1 value of 15 or lower, the free SDB suspension and IPHS-INV formulation profiles were considered to be equivalent [66].

### 3.7. In Vivo Evaluation of IPHS-INV Formulation

#### 3.7.1. Procedure

Ethics Committee approval (024-013) was obtained from Beni-Suef University. Thirty-six male Wistar rats, ranging in weight from 200 to 250 g and aged two to three months, were housed in an experimental facility that satisfied the following criteria: a 12 h light/dark cycle, humidity levels of 50 ± 5, and temperatures ranging from 20–25 °C. The rats were fed with a standard meal for one week.

#### 3.7.2. Bioavailability Studies

Using a random selection method, twelve rats were split into two sets of six. A group was given SDB orally by an oral gavage needle. Another group was given IPHS-INV formulation intratumorally. In tubes coated with EDTA, blood samples were removed from the retro-orbital plexus at various intervals throughout the course of 24 h and centrifuged. After being combined with 0.2 mL of acetonitrile, the plasma was spun at 13,000 rpm for 10 min before being allowed to evaporate. A mobile phase comprising 10% methanol, 10% distilled water, and 80% acetonitrile was used to reconstitute the residue. To estimate the SDB concentration, an isocratic separation of SDB was performed using a C-18 column (4.6 mm × 250 mm), a flow rate of 1 mL/min, and a ƛ_max_ of 254 nm [67]. Pharmacokinetic parameters such as C_max_, T_max_, t_0.5_, MRT, and AUC were measured (n = 6) using the pKSolver program. By contrasting the SDB oral suspension with the IPHS-INV formulation, the relative bioavailability was assessed.

#### 3.7.3. DMBA-Induced Skin Cancer and Management

Four sets of six rats each were randomly assigned to the remaining twenty-four rats. The dorsal skin parts of each rat were shaved using a hair clipper 48 h before the experiment began. In the control negative group, rats were given a normal diet in addition to topical applications of acetone. Following the protocol described by Bhattacharyya et al., skin cancer was induced in a further 18 rats using a tumor initiator called DMBA [58]. Randomly, the experimental skin cancer-induced rats were divided into three groups of six. In the positive control group, rats were given a croton oil transdermal acetone solution. In the free SDB group, rats were orally administered a suspension of SDB before giving a croton oil transdermal acetone solution. In the S-IPHS-INV group, rats were administered an intratumoral IPHS-INV formulation before giving a croton oil transdermal acetone solution.

#### 3.7.4. Assessment of Effectiveness of Treatment

##### Tumor Volume Measurement

Examining the tumor’s growth over time is a typical approach to determining the treatment’s effectiveness [24]. The tumor volume was monitored weekly (n = 6) using digital calipers as follows:

(8) $$\mathrm{Tumor}\;\mathrm{volume}\;=\frac{(\mathrm{width}\;\mathrm{of}\;\mathrm{tumour}\;\mathrm{mass}{)}^{2}\times \mathrm{length}\;\mathrm{of}\;\mathrm{tumour}\;\mathrm{mass}}{2}$$

##### Body Weight Measurement

Each rat’s weight (n = 6) was recorded using a delicate balance on three separate occasions: once before medication began, once weekly throughout the experiment, and once on sacrifice day [68].

##### Mortality and General Behavior

The animals were monitored for clinical symptoms and behavioral patterns every day for the dose period. This monitoring occurred before dosing, shortly after dosing, and up to 2–4 h following treatment. The mortality or lifespan of each treatment was assessed by calculating the MST utilizing Kaplan–Meier survival analysis in MedCal software [69].

##### Histopathology Study

A histological study was carried out to confirm that each therapy had the desired effect. All rats were injected intraperitoneally with a ketamine/xylazine mixture at the end of the trial. After that, cervical dislocation was used as a method of euthanasia. A 10% buffered formaldehyde solution was used to fix the tumors after their meticulous removal. Hematoxylin and eosin were used to dye thin slices of tumors (4–6 µm) in accordance with the normal methods for histopathological studies.

#### 3.7.5. Targeting Study

In order to determine the amount of SDB that remained in each organ, both the oral free SDB and IPHS-INV formulation groups had their tumor and liver tissues sliced and kept at −80 °C. The tissue slices were mixed with acetonitrile and subjected to sonication for 30 min to ensure complete release of SDB. To get rid of any undesirable contaminants, the mixture was spun in a centrifuge at 6000× *g* for a duration of 10 min and then evaporated. A mobile phase was used to reconstitute the residue. The drug content was assessed using the HPLC method.

#### 3.7.6. Toxicity Studies of IPHS-INV Formulation

##### General Behavior

The weights of the rats in the IPHS-INV formulation group and the control negative group were recorded before, during, and after sacrifice, and any behavioral changes during the trial were observed [34].

##### Evaluation of Hematological Parameters

Blood samples were taken and added to a container that contained EDTA. A gentle shaking of the sample bottle was used to combine the blood with EDTA and avoid contaminating the clothing. Quantification of serum red blood cells, white blood cells, hemoglobin, hemocytoplasmic titer, and monocytes was performed according to standard procedure [34].

##### Determination of Biochemical Parameter

The orbital method was used for blood collection, and the serum was completely separated by centrifugation. The serum SGPT, SGOT, urea, and creatinine were performed according to standard procedure [34].

### 3.8. Statistical Analysis

The information was analyzed with SPSS (version 22, Chicago, IL, USA) with a *p*-value < 0.05. To represent all data, the mean ± standard deviation (SD) was applied.

## 4. Conclusions

The objective of this investigation was to design an intratumoral in situ pH-sensitive hydrogel formulation of S-INV to make SDB more bioavailable and effective and to prolong SDB’s release so that it may be used as a treatment for basal cell skin cancer. The IPHS-INV formulation enhanced the sustainability, permeation, and bioavailability of SDB compared to the free SDB suspension by 57.4%, 6.65-fold, and 6-fold, respectively. The intratumoral IPHS-INV formulation showed no observable behavioral deficits or death and enhanced the anti-tumor activity in terms of tumor volume, body weight, and mortality compared with the positive control group. These findings support the use of intratumoral IPHS-INV formulation as a viable and effective strategy for the treatment of basal cell skin cancer.

The present study reports that IPHS-INV formulation design could significantly change the landscape for BCSC management, opening up new avenues for therapies that aim to prevent and even restore skin structure, ultimately improving patient care and advancing clinical research. It may allow for a decrease in dosage, which would reduce the probability of side effects. The benefits of IPHS-INV formulation may also apply to patients with non-small-cell lung cancer and other solid tumors, according to the results presented in this paper. But, in order to acquire human use approval, large-scale animal preclinical experiments and clinical trials must still be carried out. Additionally, the procedure for inducing skin cancer is a limitation of this study. There are substantial behavioral, genetic, and physiological differences between tumors induced by DMBA in mice and human skin cancer. Additionally, DMBA has the potential to influence the immune system and other normal cells and tissues in the tumor’s immediate vicinity. We will address these shortcomings in our upcoming article using complementary models, such as xenograft mouse models. There will be a comparative assessment of intratumoral IPHS-INV formulation and topically administered iontophoresis with subcutaneous injections of the IPHS-INV for the treatment of BCSC.

## Figures and Tables

**Table 1:** Compositions and responses of S-INV formulations.

Run	Independent Factors			Responses (n = 3)	
	Ethanol Concentration (%)	Phospholipid Concentration (%)	Cineole Concentration (%)	Entrapment Efficiency (% ± SD)	Vesicle Size (nm ± SD)
F1	5	5	1	83.39 ± 0.61	314.9 ± 4.46
F2	3	1	0.5	55.72 ± 0.71	181.2 ± 3.38
F3	5	3	0.5	61.27 ± 0.76	221.9 ± 7.21
F4	5	1	1	58.10 ± 0.51	190.1 ± 5.72
F5	5	3	1.5	74.93 ± 0.72	263.9 ± 2.56
F6	1	3	1.5	83.88 ± 0.56	280.3 ± 7.12
F7	3	3	1	72.74 ± 0.59	249.0 ± 4.53
F8	3	1	1.5	69.42 ± 0.81	222.1 ± 3.10
F9	3	5	1.5	94.54 ± 0.63	349.2 ± 3.48
F10	1	1	1	67.07 ± 0.74	211.2 ± 3.76
F11	3	3	1	72.51 ± 0.46	250.0 ± 3.93
F12	1	5	1	91.91 ± 0.47	337.5 ± 3.65
F13	3	5	0.5	80.43 ± 0.63	307.4 ± 3.94
F14	1	3	0.5	70.37 ± 0.54	238.7 ± 4.73
F15	3	3	1	72.66 ± 0.85	249.5 ± 5.72

SD: standard deviation.

**Table 2:** ANOVA analysis using Design Expert software.

Parameter	Models					
	EE			VS		
	Linear	2FI	Quadratic	Linear	2FI	Quadratic
***p-*** **value**	<0.0001	<0.0001	<0.0001	<0.0001	<0.0001	<0.0001
**F-value**	1110.88	1744.12	3702.26	491.73	227.89	839.47
**R^2^**	0.9963	0.9964	0.9965	0.9730	0.9730	0.9954
**Lack of Fit**	<0.0001	<0.0001	0.9863	<0.0001	<0.0001	0.9984
**Adjusted R^2^**	0.9956	0.9958	0.9961	0.9710	0.9687	0.9942
**Predicted R^2^**	0.9942	0.9949	0.9956	0.9674	0.9616	0.9924
**Adequate Precision**	112.0662	137.0447	186.7774	66.5953	48.4663	94.2374

**Table 3:** In vitro evaluation parameters of IPHS-INV formulation.

Formulation	Viscosity at 10 rpm (cP ± SD)	Permeation (µg/cm^2^ ± SD)	Steady State Flux (µg/cm^2^/h ± SD)	Release (% ± SD)
**Free SDB**		57.27 ± 1.33	1.57 ± 0.06	98.86 ± 0.69
**Free SDB hydrogel**	2446.40 ± 21.74	59.41 ± 1.51	1.60 ± 0.05	61.57 ± 0.97
**Optimum S-INV**	1282.35 ± 12.13	367.95 ± 6.32 ^ab^	10.7 ± 0.23 ^ab^	74.52 ± 0.54 ^ab^
**IPHS-INV**	6712.92 ± 84.26 ^a^	380.96 ± 7.57 ^ab^	11.22 ± 0.29 ^ab^	42.10 ± 0.58 ^ab^

Data denote mean ± standard deviation (SD) (n = 3). ^a^ Significance at *p*-value < 0.05 compared to free sonidegib (SDB) in situ pH-sensitive hydrogel. ^b^ Significance at *p*-value < 0.05 compared to free SDB suspension.

**Table 4:** Bioavailability study of intratumoral IPHS-INV formulation.

Pharmacokinetic Parameters	Oral Free Sonidegib (SDB)	Intratumoral IPHS-INV
t_0.5_ (h)	5.64 ± 0.27	27.54 ± 1.75 ^a^
Tmax (h)	2	4 ^a^
Cmax (ng/mL)	528.13 ± 30.68	460.96 ± 12.87 ^a^
AUC_0−α_ (ng.h/mL)	3134.81 ± 28.96	18,830.32 ± 606.18 ^a^
MRT (h)	8.18 ± 0.43	40.97 ± 2.19 ^a^

^a^ Significant at *p*-value < 0.05 compared to oral free SDB. Data denote mean ± SD (n = 6).

## Data Availability

This work’s subsequent data are included in the article.
